# High-Salt Tumor Microenvironment: Not as Bad as It Sounds, Not as Good as It Seems

**DOI:** 10.3390/cancers17121924

**Published:** 2025-06-10

**Authors:** Umer Ali, Venkataswarup Tiriveedhi

**Affiliations:** 1 Department of Biological Sciences, Tennessee State University, Nashville, TN 37209, USA; 2 Division of Pharmacology, Vanderbilt University, Nashville, TN 37232, USA

**Keywords:** cancer biology, salt, immunotherapy, T-helper cells, cytokines, tumor-initiating stem cells

## Abstract

Recent evidence suggests a high-sodium microenvironment in breast tumors. However, the exact role of this high-sodium microenvironment on tumorigenesis is unknown. Salt (sodium chloride, NaCl) is a well-known inflammatory molecule playing a significant role in various chronic ailments like cardiovascular and autoimmune diseases. Importantly, chronic inflammation is recognized as one of the major hallmarks of carcinogenesis. Breast cancer cell culture-based studies demonstrated that high-salt (HS) treatment (Δ35–50 mM NaCl) induced cancer cell proliferation. However, preclinical murine research showed reduced tumor progression kinetics in mice fed a short-term HS diet (4% NaCl diet, 0–2 weeks prior to the injection of tumor cells). Molecular studies demonstrated that the short-term HS diet induced the inflammatory activation of naïve CD4+ T cells to the Th17/Th1 anti-tumor phenotype. As human health-related adverse outcomes from HS diets usually occur as a consequence of prolonged HS intake over a period of several years, we have developed a novel chronic HS dietary murine tumor model. In this model, tumor cells are sequentially passaged (four cycles) in vivo under high-salt conditions, and tumor kinetics were analyzed in the passage-4 mice. These studies demonstrated enhanced tumor progression (pro-tumor) under chronic HS dietary conditions through the activation of tumor-initiating stem cells, along with the exhaustion of immune cells. Based on the, apparently paradoxical, evidence, we propose a comprehensive unifying hypothesis to elucidate the complex role of a high-sodium microenvironment towards tumor immune sculpting. This understanding will enable novel drug repositioning strategies, the development of unique ion channel-based anti-cancer therapeutics and promote low-salt diet intake in breast cancer patients on immunotherapy.

## Simple Summary

Breast tumors accumulate 50–70% higher sodium as compared to the surrounding soft tissue. The precise role of this intra-tumor high sodium content is unknown. Preclinical murine research showed reduced tumor progression in mice placed on a short-term high-salt diet. Molecular studies demonstrated that the short-term high-salt diet induced the anti-tumor activation of CD4+ T cells, leading to reduced tumor progression. As high-salt-related human ailments are a consequence of prolonged salt intake for years, we have developed a novel chronic high-salt dietary murine tumor model. These studies demonstrated that chronic high-salt diet intake enhanced tumor progression through the activation of tumor initiating stem cells, along with the exhaustion of immune cells. In this review article, we have discussed a novel high-salt-mediated tumor immune editing hypothesis with potential therapeutic applications.

## 1. Introduction

Breast cancer, accounting for up to 30% of all newly diagnosed female cancers in 2024, ranks as the cancer with the highest incidence among women in the United States [1]. Unfortunately, 13% of women have a lifetime risk of developing breast cancer. In past decades, the incidence of breast cancer has increased by 1% every year along with a steeper increase (1.4%) in women below 50 years old [2]. Although receptor-targeted therapeutic approaches have improved outcomes, approximately 15% of breast cancers, which lack receptors, have poor median survival and are classified as triple-negative breast cancers (TNBCs) [3].

The American Heart Association recommends a daily intake of 3.75 g of salt (sodium chloride, NaCl), which translates to 1.5 g of sodium that is required for basal metabolic needs of an average human adult [4]. An average person on a Western diet is considered to take 9 to 12 g of salt per day [5]. Salt is a well-known chronic inflammatory molecule correlated with cardiovascular, neurological, renal and autoimmune diseases [6]. While independent studies have shown a raising trend in both cancer incidence [2] and a high-salt dietary lifestyle [7], there is no direct correlation between dietary salt intake and breast cancer. Interestingly, in the human body, certain organs such as the skin and lymph nodes have a natural tendency to accumulate salt [8]. Although unknown, the pathophysiological significance of this selective accumulation of sodium in certain organs and solid tumors is an area of intense research.

The functional role of chronic inflammation has been endorsed with its acceptance as one of the hallmarks of cancer [9]. While internal factors such as germline mutations account for only up to 10% of cancers, most cancers are due to acquired external factors such as diets, obesity, pollutants and chronic infections [10]. All these external factors play an important role in chronic inflammation that impact various stages of tumorigenesis, including genomic instability, mutagenesis-mediated cellular dysplasia, metastasis, tumor immune editing, angiogenesis and chemoresistance [11]. Smoldering inflammation in the tumor microenvironment enhances pro-oncogenes (such as RAS, MYC and HER2) while potentially inhibiting tumor suppressor genes (such as p53, BRCA1 and PTEN), resulting in genomic instability and tumorigenesis [12,13]. The chronic inflammatory tumor microenvironment induces aberrant molecular signals (such as reactive oxygen/nitrogen species), leading to signaling pathways such as JAK/STAT and NF-kB-mediated mechanisms that lead to immune exhaustion and tumor progression [14]. Among various chemicals, salt (sodium chloride) is a well-known inflammatory agent and arguably an understudied aspect in cancer biology [15]. In this communication, we will review the mechanistic role of a high-sodium microenvironment in tumorigenesis and tumor immune editing.

## 2. High-Sodium Tumor Microenvironment

Nagy et al., in as early as 1981, using energy-dispersive X-ray microanalysis on freeze dried biopsies obtained from human urogenital cancers, had shown a five-fold increase in the intra-tumor sodium-to-potassium concentration ratio ([Na^+^]/[K^+^]) as compared to the normal human urogenital epithelium [16]. Hurter et al. (1982), using a similar quantitative analytical approach, have reported an increased intratumoral [Na^+^] in rat glioma models [17]. Around the same time (in the early 1980s) Sparks et al. had demonstrated that amiloride, a potassium-sparing diuretic which inhibits intracellular sodium influx, reduced tumor growth in murine hepatoma and mammary adenocarcinoma models [18,19]. Recent evidence from multiple research groups has shown a high intratumoral sodium concentration as compared to the peritumoral tissue. The semi-quantitative measure of tissue [Na^+^] by sodium magnetic resonance imaging (Na^23^-MRI) in human breast cancer patients demonstrated a 30–70% higher intratumoral [Na^+^] [20,21,22,23]. A similar increase in intratumoral [Na^+^] was noted in murine models of glioma and prostate cancer [24,25].

Interestingly, Soll et al., utilizing inductively coupled plasma optical emission spectrometry (ICP–OES), have shown higher intratumoral [Na^+^] in fresh tissue biopsies obtained from breast cancer patients [26]. Currently, due to instrumental limitations, it is difficult to reliably measure the differences in intracellular (IC) and extracellular (EC) ionic concentrations within the tumor tissue. Therefore, the tumor [Na^+^] is taken as a weighted average of the IC and EC components. There is very limited evidence determining the impact of a salt-modulated diet on the tumor [Na^+^]. In agreement with others, studies in our laboratory utilizing live in vivo Na^23^-MRI in preclinical murine breast cancer models demonstrated a 50–70% increase in [Na^+^] [27]. Further, quantitative analyses of intratumoral electrolyte concentrations have shown that a high-salt (HS) diet further increased the [Na^+^] by 25% as compared to tumors in the regular-salt diet cohort and up to a 100% increase as compared to contralateral mammary fat pad tissue in the HS diet cohort [27]. These data clearly suggest that dietary salt impacts the intra-tumor [Na^+^]. Our studies are in good agreement with preclinical Na^23^-MRI data in breast tumor models by James et al. (2022), where they have further performed ex vivo studies demonstrating a high IC [Na^+^] [28,29].

The pathophysiological role of intratumoral [Na^+^] is a topic of great interest and, also, unfortunately is an understudied field in cancer biology. Regular cellular processes result in high [K^+^] in the IC compartment, while the [Na^+^] is high in the EC compartment [30]. Although the reasons for sodium accumulation in the tumors are unknown, a probable explanation could be due to heavy loads of anionic lysine-rich extracellular matrix proteins in solid tumors resulting in the accumulation of cationic sodium ions [31]. In our previous studies, it was interesting to note that there was increased intratumoral [K^+^] in murine breast tumor models fed on a low-salt (LS) diet as compared to peritumoral [K^+^] in the same cohort [27]. More molecular and biophysical studies are needed to ascertain the exact role of a low-salt diet-mediated [K^+^] increase in the tumor microenvironment. Also, the differences between various solid organ tumors might have varying impact on the intratumoral [Na^+^]. While increased edematous extracellular fluid is well-known to be associated with gliomas, it is uncommon with other tumors such as breast cancers [21,32]. Following chemotherapy is gliomas, it was observed that there is a further increase in edema along with an increase in tumor [Na^+^] [33,34]. Conversely, following chemotherapy, in breast tumors, increased edema is associated with a decrease in tumor [Na^+^] [35].

In human tissues, the extracellular (EC) fluid [Na^+^] is approximately ten times higher than the intracellular (IC) compartment [36]. While the sodium concentration in extracellular fluid [ECF-Na^+^] ranges from 135 to 145 mM, the intracellular fluid sodium concentration [ICF-Na^+^] is 5–15 mM [37]. This difference is actively maintained by ATP-dependent Na^+^/K^+^ pumps [38]. In general, the cell membrane resists the concentration-dependent free movement of sodium ions between the IC and EC compartments. This ten-fold ionic concentration gradient across the membrane caused by ATP-dependent active Na^+^/K^+^ exchange pumps results in the resting membrane potential [39]. This Na^+^/K^+^ ATPase consumes up to 25% of all ATP generated by the cell, and it could be as high as 70% in the brain [40]. For every ATP used by the Na^+^/K^+^ ATPase, three Na^+^ are pumped out of the cell while two K^+^ are moved into the cell. This Na^+^/K^+^ exchange pump is shown to have a higher affinity to intracellular sodium ion sensing over potassium ions [41]. Importantly, the salt-inducible kinase (SIK) is shown to modulate the Na^+^/K^+^ exchange pump [42]. An enhanced SIK expression is correlated with the downregulation of the Na^+^/K^+^ ATPase, probably resulting in increased IC [Na^+^] in breast tumors [43]. On the other hand, the downregulation of Na^+^/K^+^-ATPase functionality could be due to enhanced Warburg metabolism leading to decreased ATP production [44].

We have performed a binary phospho-proteomic analysis to determine the molecular changes induced by high salt in the tumor microenvironment. With this approach we identified a unique salt-sensitive kinase, SIK3 (salt-inducible kinase-3), and a serine/threonine kinase, which has shown specific phosphorylation at site ser-493 following treatment with NaCl (Δ0.05 M) [45]. Dose–response studies with NaCl on breast cancer cell lines demonstrated that high salt along with a sub-effective dose of the pro-inflammatory cytokine IL-17 (0.1 nM, similar to smoldering inflammation in the tumor) induced cancer cell proliferation and the production of inflammatory reactive oxygen and nitrogen species (ROS/RNS) [46,47]. The SIK protein family has been shown to play an important role in various metabolic, inflammatory, hormonal and cancer pathways [48]. Enhanced SIK expression is reported in breast, lung, ovarian and prostate cancers [49]. Importantly, cell cycle analysis demonstrated that SIK3 induced the release of G0/G1-phase arrest, leading to cell proliferation [45]. Further, our studies confirmed that both the enhanced expression and phosphorylation of SIK3 were specifically stimulated by a high salt concentration while equivalent concentrations (both 0.05 and 0.1 M) of mannitol or sucrose (osmotic experimental controls) did not induce a similar response, suggesting a salt-mediated response over a potential-enhanced osmotic response. It is important to note that equivalent ionic control cannot be used, as the other salts in the same group (LiCl, KCl, RbCl and CsCl; group I alkali metals) were cytotoxic at just 5–10 mM doses. Further, orthotopic tumors induced with the intramammary injection of MCF-7 (MCF-7 cells passaged ex vivo with Δ0.05 M NaCl for eight passages) breast cancer cells into immunodeficient Nu/J mice demonstrated 1.2–1.5-fold enhanced tumor progression as compared to MCF-7 tumors [50]. Further, reduced tumor progression was noted in mice injected with shRNA-based SIK3 knock-out (SIK3-KO)-MCF-7 cells. The high-salt passaged MCF-7 cells were shown to be resistant to paclitaxel-induced cytotoxicity as there was enhanced expression of the chemoresistance factor P-glycoprotein.

**Figure 1:**
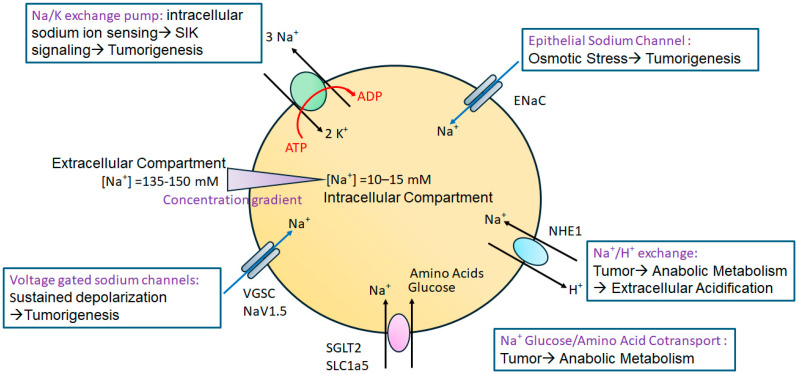
Role of various sodium channels in high-salt-mediated tumorigenesis. The Na^+^/K^+^ exchange pump, through its intracellular sodium-sensing domain, detects increased intracellular sodium, leading to SIK signaling-mediated cancer cell proliferation and metastasis. ENaC controls the concentration gradient-mediated movement of sodium ions, and high-salt-induced osmotic stress activates ERK1/2-mediated inflammatory signaling and cancer cell proliferation. VGSCs through am increased intracellular sodium concentration induces the sustained depolarization of the cell, leading to multiplication and tumorigenesis. The sodium cotransporter channels SGLT2 and SLC1a5 allow the intracellular transport of glucose and amino acids needed for anabolic metabolism during cancer cellular growth. NHE1 exchanger channel activity causes elevated intracellular sodium concentrations and [H^+^] efflux, resulting in low extracellular pH favoring invasion and metastasis through the extracellular matrix.

The sustained depolarization of cells was shown to induce mitotic activity and tumorigenesis [51]. Increased IC [Na^+^] would reduce the sodium gradient across the cell membrane, resulting in membrane depolarization [29]. To fuel anabolic metabolism, cancer cells would acquire increased capacity for glucose intake [52]. Indeed, the diagnostic tool positron emission tomography (PET) scan utilizes the enhanced cellular ability of cancer cells to uptake 2-deoxy-2-[^18^F]-fluoro-d-glucose selectively to detect metastatic nodules [53]. The Na^+^–glucose cotransporter (SGLT2) facilitates this import of glucose along with sodium ions. Increased SGLT2 expression was shown in breast, lung, prostate and pancreatic cancers [54]. The activation of voltage-gated Na^+^ channels (VGSCs) also induces the depolarization of the cell membrane (Figure 1). Increased expression of VGSCs was noted in several solid organ tumors [55]. However, as VGSCs are fast channels, the failure of these channels to maintain their inactivated state will cause persistent Na^+^ influx, resulting in sustained depolarization [56]. Aberrant VGSC expression is correlated with cell motility and metastasis [57]. Mechanistically, VGSC-mediated sustained depolarization was shown to activate GTPase/Rac1 molecules, causing the branching of the actin cytoskeleton, leading to lamellipodia formation and resulting in cancer cell metastasis [58,59,60]. Another mechanism by which the VGSC is considered to mediate metastasis is by the downstream activation of NHE1 (Na+/H+ exchanger) channels that lead to H^+^ extrusion from cancer cells, resulting in the extracellular acidification of the tumor microenvironment [61,62]. However, the exact role of sodium influx through VGSCs and H^+^ extrusion culminating into tumor metastasis has yet to be determined. Acidic pH is well-recognized in the extracellular tumor microenvironment [63]. This phenomenon can also be explained by enhanced glycolysis and decreased mitochondrial oxidative metabolism, leading to the increased synthesis of acidic anabolic macromolecules needed for cell proliferation due to a phenomenon called the Warburg effect. There is an intricate connection between cellular pH regulation and sodium ionic homeostasis. Acid-sensing sodium channels such as ENaC (epithelial sodium channel), NHE1 and NBC (sodium-bicarbonate cotransporter) have been shown to be upregulated in breast cancers [64,65,66]. More studies are needed to comprehensively determine the role of these various sodium channels in breast tumorigenesis.

## 3. Is a High-Salt Tumor Microenvironment Bad?

While the high-salt treatment promoted tumor cell proliferation, there are some polar opposite results emanating from research studying the impact of the HS diet in immunocompetent preclinical murine models. Evidence from multiple research groups, including ours, demonstrated that the HS diet reduced tumor progression in preclinical models. Studies by Willebrand et al. have demonstrated that there was decreased tumor kinetics in both B16F10 melanoma- and Lewis lung carcinoma-based murine tumor xenografts when the mice were fed an HS diet (a 4% NaCl diet supplemented by 1% NaCl water) [67]. In these studies, they found that the HS diet blocked the functionality of myeloid-derived suppressor cells (MDSCs), leading to anti-tumor effector adaptive immune responses. In the tumor microenvironment, MDSCs were shown to inhibit the anti-tumor impact of adaptive immune responses, specifically the tumor elimination capability of CD8+ and CD4+ T cells. Similarly, He et al. have also shown that the HS diet induced an anti-tumor effect through the differentiation of MDSCs to macrophages [68]. It is important to note that our studies confirm that while there is high sodium accumulation in breast tumors, the HS diet further enhanced the intratumor [Na^+^] by 40% [27]. The high-salt treatment (Δ35–50 mM NaCl) is shown to induce the pro-inflammatory (Th17 and Th1) phenotype switch of naïve CD4+ T cells. Molecular mechanistic studies demonstrated that serum glucocorticoid kinase (SGK1) and osmo-sensitive transcription (TonEBP/NFAT5) played an important role in mediating this pro-inflammatory switch of adaptive immune cells [69,70]. In preclinical models, the HS diet had an onerous impact on disease progression in experimental autoimmune encephalitis (EAE, a preclinical model for multiple sclerosis) and graft-versus-host disease (GvHD) [71,72]. Taken together, the evidence from short-term HS diet studies suggests that an HS diet could play a positive role by inhibiting tumor growth.

In line with the above evidence, our studies have shown that the HS diet induced tumor regression through the inflammatory activation of CD4+ T cells to the Th1 and Th17 phenotypes [27]. Also, we have not noticed significant differences in breast tumor progression kinetics between the regular-salt and low-salt diet cohorts. In stark contrast to all the above-mentioned studies, Chen et al. demonstrated that the HS diet enhanced tumor progression and lung metastasis in a spontaneous MMTV-PyVT-based preclinical breast tumor model [73]. These apparently opposite results could be due to differences between spontaneous versus xenograft-based murine tumor models which require further investigation. However, it is more important to note the differences in the duration of the HS dietary treatment conditions in various research reports. Willebrand et al. and He et al. introduced their xenograft tumor models to an HS diet 0–2 weeks prior to the injection of cancer cells [67,68]. In our studies we introduced the HS diet 2 weeks prior to the orthotopic injection of breast cancer cells. This suggests that a short-term HS diet activates effector immune responses, leading to the initial elimination phase of tumor immune editing and ultimately resulting in an anti-tumor response. However, Chen et al. introduced mice to the HS diet at 4 weeks of age and followed them for 12 weeks to verify spontaneous tumor development and progression [73]. These results suggest that in spontaneous murine models, tumorigenesis could be a consequence of cancer cells overcoming the initial step of high-salt-mediated immune elimination and reaching the later stages of tumor immune surveillance, namely the equilibrium and escape phase of immune editing that ultimately results in a pro-tumor response. More detailed immunological mechanistic studies would be needed to characterize the immune responses involved in the HS diet-mediated pro-tumor responses observed in the spontaneous breast tumor model.

## 4. Is a High-Salt Tumor Microenvironment Good?

**Figure 2:**
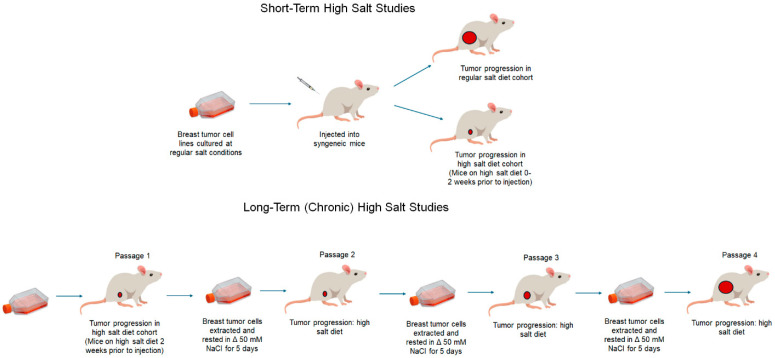
Short-term and long-term (chronic) high-salt dietary tumor models. In short-term HS diet studies, tumor cells are cultured under regular culture conditions. Mice will be placed on the HS diet for 0–2 weeks following which syngeneic tumor cells will be injected to induce tumor growth for the analysis, and various cellular and immunological studies will be performed. In chronic (long-term) HS diet studies, syngeneic tumor cells will be injected into mice placed on the HS diet for 2 weeks prior to injection. Following the tumor growth end point, the tumors will be explanted and digested to obtain tumor cells. These tumor cells will be cultured in HS (Δ50 mM NaCl) conditions for 5–7 days and later injected into new syngeneic mice placed on the HS diet 2 weeks prior to injection. This cycle is repeated 4 times. At the end of the fourth cycle (passage 4), tumor growth analysis and various cellular and immunological studies will be performed.

A possible reason for these apparently conflicting outcomes in tumor progression following dietary salt modulation could be due to a lack of a consensus on the duration of the HS diet needed for tumor immune editing. Further, the short-term HS diet utilized in pre-clinical models is not a realistic representation of human settings, wherein the HS diet is a long-term lifestyle adopted over a period of several years. Further, while the short-term HS diet could lead to the activation of anti-tumor effector immune responses, on the other hand, the long-term HS diet could cause immune exhaustion. To verify these potential limitations of the short-term HS diet, we have developed a novel chronic HS diet preclinical murine orthotopic tumor model (Figure 2) [74]. It is important to note the challenges of developing a chronic HS dietary preclinical murine tumor model. As per IACUC (Institutional Animal Care and Use Committee) protocols on the humane treatment of murine models, the tumors should not grow to more than 1.5 cm in diameter. Furthermore, in mice carrying a tumor burden for a prolonged amount of time (up to 6 months tumor burden), there will be unacceptable ulceration at the tumor site and inflammatory sickness. To address these experimental challenges, we have developed a novel chronic (long-term) HS dietary murine tumor model. Briefly, to generate this chronic HS diet model, we performed sequential passage of tumor cells in mice under HS dietary conditions (passage 1, P1, to passage 4, P4). The mice were placed on an HS diet (4% NaCl) for 2 weeks prior to the injection of syngeneic murine breast cancer cells and later continued through the tumor-bearing phase. The tumors from these mice (P1) were harvested on day 28, and tumor cells were collected by collagenase digestion and rested in a cell culture incubator for 5 days under high-salt culture conditions (Δ 50 mM NaCl). These high-salt-cultured P1 passaged tumor cells are injected into mice kept on a high-salt diet (as mentioned above). This process is repeated for a total of four cycles (P1 to P4). Further, immune and metabolic analysis demonstrated that the chronic HS diet induced tumor-initiating stem cells and Warburg-like glycolytic metabolism which are needed for the synthesis of anabolic metabolites for tumor cell growth and proliferation. Importantly, the analysis for tumor-infiltrating T lymphocytes in P4 tumors demonstrated enhanced expression of the immune checkpoint inhibitor CTLA4, while those in P1 demonstrated a higher frequency of the effector Th17/Th1 phenotype. These data suggest that the long-term HS diet as a lifestyle practice could be potentially deleterious to cancer patients.

## 5. Unifying Theory to Explain the Role of High Salt in Tumor Immune Sculpting

**Figure 3:**
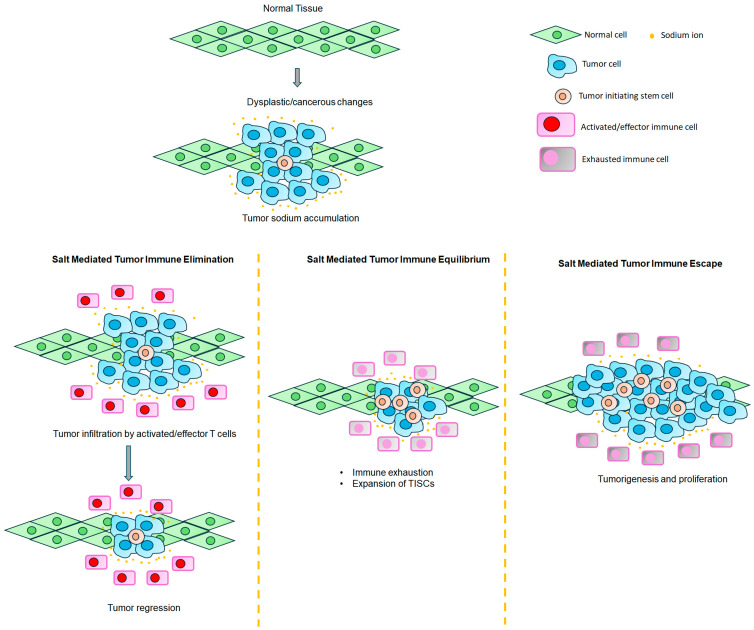
Unifying theory to explain the role of a high-sodium microenvironment in tumor immune editing. High sodium accumulation in the breast tumor tissue exerts an initial protective role through the elimination of transformed cancerous cells by the activation of innate and adaptive immune responses. Specifically, during this initial elimination phase, the high salt concentration in the tumor microenvironment activates naïve CD4+ T cells to the effector TH1/Th17 phenotype, along with the phenotypic switch of M-MDSCs to the MΦ1 anti-tumor phenotype. In the second phase, there is immune exhaustion and the activation of TISCs, during which the tumor enters an immune equilibrium phase due to the immunosuppressive tumor microenvironment. In the third (final) phase, the tumor evolves and escapes hostile effector immune responses along with the expansion and tumor cell transformation of TISCs, thus resulting in tumor proliferation.

The role of the immune system in the dual function of cancer control and cancerous transformation is broadly termed as cancer immune editing. Based on evidence from multiple lines of research, we have attempted to develop a unifying theory to explain the role of salt in tumor immune sculpting (Figure 3). A high sodium concentration in the tumor microenvironment is strongly suggested to play a dual role in host protection and tumor-sculpting actions resulting in tumor progression. We propose a high-salt-mediated cancer immune editing theory to explain the paradoxically opposite role of a high-salt microenvironment in shaping tumor destiny in three temporally separated phases through the modulation of immune responses.

*Salt-mediated tumor immune elimination*: In the initial phase, the high-sodium tumor microenvironment induces the elimination of transformed cancerous cells by various myeloid and lymphoid immune system components. Studies by Willebrand et al., utilizing the B16F10 syngeneic melanoma transplantation and Lewis lung carcinoma (LLC) models, showed a significant decrease in tumor growth in mice fed a short-term HS diet (mice placed on the HS diet 2 weeks before the injection of syngeneic tumor cells) [67]. Cytokine gene expression analysis showed increased tumor transcript levels of *Tnf*-α and *Ifn*γ. Further, a higher frequency of CXCR3+ CD4+ T cells in the draining lymph nodes was noted in HS-fed tumor-bearing mice. It is to be noted that the CXCR3 chemokine receptor is known to play an active role in immune cell recruitment [75]. Along with adaptive immune responses, the HS diet has been shown to modulate peripheral innate immune responses, particularly the monocyte/macrophage populations. Immunological studies by this group have suggested that the HS diet antagonized the suppressive function of MDSCs, potentially leading to an enhanced anti-tumor impact by Mφ1 macrophages.

Similarly, studies by He et al. have also demonstrated that the short-term HS diet (mice placed on an HS diet at the time of injection of syngeneic tumor cells) inhibited the tumor progression in the B16F10 melanoma and 4T1 mammary tumor models [68]. Further, cytokine analysis by this research group showed that the HS diet increased the expression of pro-inflammatory cytokines such as, IL-12p40, ICAM-1, IFN-γ and TNF-α, along with a decreased expression of IL-6, IL-10 and GM-CSF. Further, the HS diet significantly reduced the peripheral and tumor-infiltrating frequency of M-MDSCs (CD11b+Gr-1+ Ly-6C+) with minimal change in the PMN-MDSCs (CD11b+Gr-1+ Ly-6G+) frequency. This reduced frequency of M-MDSCs was postulated to be due to the phenotypic differentiation of M-MDSCs to the anti-tumor macrophage phenotype. However, both groups observed some conflicting results when studying the impact of the short-term HS diet in immunodeficient murine tumor models. While Willebrand et al. saw decreased tumor progression in Rag2^−/−^ immunodeficient mice fed on the HS diet, suggesting an apparent lack of the role of T cells in HS diet-mediated tumor inhibition, He et al., utilizing BALBc^nu/nu^ mice, showed a lack of difference in tumor progression kinetics between the HS diet and RS diet cohorts, suggesting a critical role of T cells in HS diet-mediated tumor inhibition. This divergence in data could be due to the differences in murine models, wherein BALBc^nu/nu^ mice lack the thymus, leading to a lack of T-cells, while in Rag2^−/−^ mice there is improper development of T cells. Further studies optimizing the immunodeficient murine models are needed to address this discrepancy.

Studies from our laboratory utilizing the Py230-C57Bl/6 breast tumor model demonstrated that the short-term HS diet (mice placed on an HS diet 2 weeks before the injection of syngeneic tumor cells) reduced tumor progression [27]. Further, immunological studies demonstrated that the short-term HS diet induced the inflammatory activation of naïve CD4+ T cells to the Th1 and Th17 phenotypes. This was associated with enhanced tumor microenvironment expression of inflammatory cytokines, such as IFNγ and IL-1β. Mechanistic studies have demonstrated a critical role of the osmotonic transcription factors TonEBP/NFAT5 (Nuclear Factor of Activated T-cells 5) in inducing HS diet-mediated inflammatory activation of CD4+ T cells. Ex vivo studies in our laboratory have also demonstrated that the cell culture of CD4+ T cells obtained from tumor-infiltrating and tumor-draining lymph nodes under HS treatment conditions induced the expansion of the Th1 and Th17 CD4+ T cell phenotypes which upon autologous infusion into mice induced tumor regression [70]. Similarly, studies by Scirgolea et al. have also demonstrated the anti-tumor effector activation of CD8+ T cells by HS in vitro cell culture treatment conditions [76]. All these data strongly suggest the direct role of HS in the initial elimination phase of tumor immune editing.

*Salt-mediated tumor immune equilibrium*: In general, the molecular triggers which induce tumor immune equilibrium are poorly enunciated. There is minimal evidence suggesting an infliction point with a switch from pro-inflammatory IL-12- and IFNγ-mediated tumor immune elimination to IL-23- and IL-10-mediated tumor immune equilibrium [77,78]. Further, during this equilibrium phase, tumors are considered to be in a state of dormancy. Chronic inflammatory stimulation of T cells lead them to a state of immune exhaustion, leading to the expression of markers such as PD1 on CD4+ T cells [79]. As the tumor immune equilibrium phase is temporally placed after the tumor immune elimination phase, utilizing regular short-term HS diet models will be insufficient to characterize this phenomenon. As mentioned earlier, to study these temporally spaced phenomena, we have developed a novel chronic HS diet model, wherein we have performed temporally spaced sequential passage of tumor cells (four passages, P1 to P4) in the HS diet-fed mice. These studies have demonstrated that there was an increase in expression of exhaustion markers in passage 3 (P3) of our chronic HS diet tumor-bearing murine model [74]. From P1 to P4, there was up to a 3-fold increase in CTLA4 and a 2-fold increase in the TIM3, LAG3 and PD1 (individually) exhaustion markers, along with a 3-fold decrease in granzyme B and an 8-fold decrease in IFNγ inflammatory markers, suggesting an immunosuppressive tumor microenvironment.

Tumor-initiating stem cells (TISCs) play an important role in tumor dormancy and recurrence and treatment resistance [80]. Thus TISCs, along with PD1, could be potentially used as marker for tumor immune equilibrium. In both chronic HS diet murine breast tumor models (Py230-C57Bl/6J and 4T1-BALB/cJ), we observed up to an 8-fold increase in CD44+CD24- TISCs from P1 to P4 [74]. Further gene expression analysis for TISC markers demonstrated that, from P1 to P4, there was an up to 6-fold increase in Cadherin1, a 10-fold increase in Snail2, an 8-fold increase in Aldh1A1 and a 6-fold increase in ITGA6 transcript levels. These data demonstrated that the chronic HS diet induced the tumor immune equilibrium phase following initial tumor immune elimination.

*Salt-mediated tumor immune escape*: In the final phase of tumor immune editing, the tumor overcomes the host’s effector immune responses and also skews the tumor microenvironment with an immunosuppressive signature. Our chronic HS diet tumor models demonstrated enhanced tumor progression kinetics in P4 tumor-bearing mice fed the HS diet. Further, mechanistic studies revealed an important role of the TGFβ/CD80 signaling mechanism that lead to the expansion of TISCs and tumorigenesis along with the CD80/CTLA4-mediated immunosuppressive tumor microenvironment [74]. Taken together, these data clearly show that a high-salt tumor microenvironment plays a crucial role in tumor immune sculpting.

Future studies are required to determine the potential reasons for the selective deletion of T-cell clones from lymph nodes and thereby the ablation of memory responses against tumor antigens. It is important to explore the role of TCR affinity of nascent T cells to tumor antigen epitopes as it can result in the deletion of certain clones involved in tumor immune elimination, while other clones progress to equilibrium and escape. During tumor immune sculpting, it is imperative to expect genomic instability that results in the evolution of new tumor variants to overcome the hostile host’s effector immune responses. Further genomic studies are required to identify the molecular mechanisms leading to tumor variance, the suppression of the MHC class I pathway and tumor antigen mutagenesis.

## 6. Clinical Applications and Future Directions

Cell-based immunotherapy offers a novel anti-cancer approach. Several clinical trials with varying levels of success have been adopting chimeric tumor-associated antigen (CAR-T cells) and autologous transfer of activated T-cell strategies [81,82]. However, a major limitation is the generic nature of these approaches. A more personalized therapeutic strategy would be needed to specifically target the unique cancer genotype specific to a given patient. By utilizing a preclinical murine model, we have developed a novel ex vivo expansion model of tumor-primed effector CD4+ T cells [70]. We have isolated CD4+ T cells from the tumor-infiltrating immune cells and tumor-draining lymph nodes of tumor-bearing mice. These CD4+ T cells were ex vivo expanded with the treatment of Δ35 mM NaCl, anti-CD3/CD28, IL-2 and IL-7 along with heat-killed tumor cells. This ex vivo expansion induce a Th1/Th17 phenotype switch of CD4+ T cells which when injected into mice caused reduced tumor progression compared to the respective experimental controls. Molecular mechanistic studies using transgenic mice have demonstrated a critical role of NFAT5 in this high-salt-mediated tumor-specific effector phenotype switch. These proof-of-concept studies require validation with appropriate clinical trials to verify its possibility as a viable personalized therapeutic option in cancer patients.

Cardiotoxicity and immune-related adverse events (irAEs) are common side effects of immune checkpoint inhibitor (ICI) therapy leading to treatment-related mortality. Our studies demonstrated that a high-salt diet in preclinical breast tumor models on ICI therapy demonstrated enhanced systemic irAE responses along with inflammatory damage of lungs [27]. Interestingly, breast tumor-bearing mice on anti-CTLA4 therapy when placed on a low-salt diet demonstrated decreased peripheral circulatory levels of inflammatory cytokines (IFNγ and IL-1β) and reduced lung infiltrating of inflammatory immune cells along with reduced tumor progression. However, it is also important to note that the majority of cancer chemotherapies result in cellular lysis and circulating electrolyte imbalance. Therefore, more human studies are required to determine the potential beneficial impact of a low-salt diet along with its impact on circulating electrolyte levels in cancer patients on ICI therapy.

It still unclear on the exact reasons for high sodium accumulation in tumors. However, tissue sodium measurements require a sample with at least 50–70 μL of tumor tissue volume, which might not be feasible. Sodium magnetic resonance imaging (^23^Na-MRI) offers a non-invasive semi-quantitative diagnostic technique to measure the tumor sodium concentration [83]. Due to the impact of various sodium ion channels on cellular functionality and tumorigenesis (described above), an elevated tissue sodium concentration was noted in malignant breast tumors as compared to benign and healthy breast tissues. Radiological research studies have strongly suggested an important diagnostic and prognostic role of ^23^Na-MRI in breast cancer [23]. Studies by Zaric et al. have shown the utility of 7.0-T MRI in the quantitation of breast tumor tissue sodium concentration with a predictive role in the favorable outcomes with neoadjuvant chemotherapy [84]. Cellular proliferation is one of the major tumor characteristics which determines the chemotherapeutic outcome. It is important to note that the MRI technique provides a diffusion-weighted average in imaging the tumor tissue sodium concentration. However, it would not be possible to differentiate intracellular versus extracellular accumulation of sodium. In spite of this limitation, ^23^Na-MRI could become an invaluable non-invasive tool to determine the therapeutic outcome and prognosis in breast cancer patients. More clinical studies are needed to determine if sodium accumulation corelates with the benign versus malignant nature (include grade/stage) of the tumor.

A correlation has been shown between poor outcomes in breast cancer and the expression of sodium channels such as ENaC, NaV1.5, VGSC, etc. More studies are needed to ascertain the potential application of these molecular targets as biomarkers in cancer treatment [85,86,87]. Importantly, renin–angiotensin–aldosterone system (RAAS) inhibitors widely used in hypertension treatment have been shown to have a beneficial effect when used with ICI therapy in cancer patients [88]. While sodium channel-targeting drug inhibitors have been utilized as anesthetics and also in cardiac and neurological diseases, there is limited direct clinical evidence on their role in cancer therapy. Experimental and observational clinical data suggest that VGSC inhibition-based local anesthetic treatment (lidocaine) at the time of surgical tumor removal resulted in up to a 57% lower recurrence rate as compared to general anesthesia and morphine [89]. Similarly, sodium channel-inhibiting anti-epileptic drugs (such as valproic acid and lamotrigine) have been shown to play an anti-metastatic role in cancer prognosis, warranting their potential use as anti-cancer adjuvants [85,90]. The antiarrhythmic drug ranolazine, a VGSC inhibitor used in cardiac diseases, is shown to inhibit metastasis in preclinical breast and prostate tumor models [91]. Results from ongoing clinical trials (NCT01916317 and NCT02786329) will shed more light on the potential application of sodium channel inhibitors in cancer outcomes. More clinical trials are also needed to study the role of these channel inhibitors in various cancer therapies.

## 7. Conclusions

In conclusion, the role of salt in the tumor microenvironment is an interesting and understudied area in cancer biology. More research in this direction would pave the way for the identification of novel biomarkers with an impact on cancer prognosis. A low-salt diet seems like a viable lifestyle modification recommendation for cancer patients, especially for those on ICI therapy. More epidemiological studies are needed to provide recommendations on the optimal dietary salt intake in cancer patients. In this review, we have put forward a unifying theory to explain the role of the high-sodium tumor microenvironment on tumor immune sculpting. Further research in this direction could enable the development of novel anti-cancer drug combinations with sodium channel inhibitors.

## Abbreviations

HS	High salt
IC	Intracellular
EC	Extracellular
Na^+^	Sodium ion
[Na^+^]	Sodium ion concentration
LS	Low salt
RS	Regular salt
TISC	Tumor initiating stem cell
VGSC	Voltage gated sodium channel
SGLT2	Sodium glucose cotransporter 2
